# Identification and Characterization of the Very-Low-Density Lipoprotein Receptor Gene from *Branchiostoma belcheri*: Insights into the Origin and Evolution of the Low-Density Lipoprotein Receptor Gene Family

**DOI:** 10.3390/ani13132193

**Published:** 2023-07-04

**Authors:** Yunpeng Cao, Haili Wang, Ping Jin, Fei Ma, Xue Zhou

**Affiliations:** 1 School of Chemistry and Biological Engineering, Nanjing Normal University Taizhou College, Taizhou 225300, China; c1845488557@163.com; 2 Laboratory for Comparative Genomics and Bioinformatics & Jiangsu Key Laboratory for Biodiversity and Biotechnology, College of Life Science, Nanjing Normal University, Nanjing 210046, Chinajinping@njnu.edu.cn (P.J.); mafei01@tsinghua.org.cn (F.M.)

**Keywords:** lancelet, transmembrane protein, molecular phylogeny, full-length cDNA

## Abstract

**Simple Summary:**

The low-density lipoprotein receptor (LDLR) family plays crucial roles in lipid metabolism, but this family has not been investigated in ancient chordates (protochordates) to date. We identified and characterized a new LDLR family member in a protochordate—*Branchiostoma belcheri*. Additionally, we investigated the evolutionary process and molecular function in this gene family. Our work fills a research gap in the LDLR family in protochordates and provides new insights into the origin and evolution of chordate LDLRs.

**Abstract:**

Low-density lipoprotein receptors (LDLRs) are a class of cell-surface endocytosis receptors that are mainly involved in cholesterol homeostasis and cellular signal transduction. Very-low-density lipoprotein receptors (VLDLRs), which are members of the LDLR family, have been regarded as multi-function receptors that fulfill diverse physiological functions. However, no *VLDLR* gene has been identified in protochordates to date. As a representative protochordate species, amphioxi are the best available example of vertebrate ancestors. Identifying and characterizing the *VLDLR* gene in amphioxi has high importance for exploring the evolutionary process of the LDLR family. With this study, a new amphioxus *VLDLR* gene (designated *AmphiVLDLR*) was cloned and characterized using RACE-PCR. The 3217 nt transcript of the *AmphiVLDLR* had a 2577 nt ORF, and the deduced 858 amino acids were highly conserved within vertebrate VLDLRs according to their primary structure and three-dimensional structure, both of which contained five characteristic domains. In contrast to other vertebrate VLDLRs, which had a conserved genomic structure organization with 19 exons and 18 introns, the *AmphiVLDLR* had 13 exons and 12 introns. The results of a selective pressure analysis showed that the *AmphiVLDLR* had numerous positive selection sites. Furthermore, the tissue expression of *AmphiVLDLR* using RT-qPCR showed that *AmphiVLDLR* RNA expression levels were highest in the gills and muscles, moderate in the hepatic cecum and gonads, and lowest in the intestines. The results of the evolutionary analysis demonstrated that the *AmphiVLDLR* gene is a new member of the VLDLR family whose family members have experienced duplications and deletions over the evolutionary process. These results imply that the functions of LDLR family members have also undergone differentiation. In summary, we found a new *VLDLR* gene homolog (*AmphiVLDLR*) in amphioxi. Our results provide insight into the function and evolution of the *LDLR* gene family.

## 1. Introduction

Very-low-density lipoprotein receptors (VLDLRs) are classified into the superfamily of transmembrane-protein low-density lipoprotein receptors (LDLRs). The *VLDLR* gene was initially discovered as its homologous gene (*LDLR*) in rabbits [1] and was later identified in many animals including nematodes [2], fish [3,4], birds [5,6], and some other mammals [7,8]. LDLRs bind to apo-E- and apo-B-containing lipoproteins, whereas VLDLRs preferentially interact with triglyceride-rich lipoproteins and apo-E [9]. In humans, *VLDLR* mRNAs are expressed in various tissues such as adipose, heart, and muscle tissues but are expressed very little in the liver [8,10]. The ligand specificity and tissue distribution of the VLDLR showed that the receptor may have critical functions in fatty acid metabolism. Similar to LDLR family members, the VLDLR protein consists of four or five characteristic regions, including a C-terminal intracellular domain, a single-transmembrane-spanning segment or transmembrane domain, optional O-linked glycosylation domain, epidermal growth factor (EGF) precursor homology domain, and an N-terminal ligand-binding domain [11]. Although each structural domain of the LDLR and VLDLR proteins exhibits a unique homology to some extent, the VLDLR has eight cysteine-rich repeat regions in the ligand-binding domain, whereas LDLRs have seven. VLDLRs are classified into two subtypes: type I VLDLRs contain five functional domains and type II VLDLRs lack the O-linked sugar fragment encoded by the 16th exon and are prominent in all tissues except muscle. Type I VLDLRs are principally expressed in muscles and the heart with active lipid metabolism, whereas type II VLDLRs are mainly expressed in the kidneys, spleen, adrenal gland, and testis [12].

These LDLR family members are composed of a large variety of receptors. Together with LDLRs, other family members also include very-low-density lipoprotein receptors (VLDLRs), apolipoprotein E receptor-2 (ApoER2), LDLR-related protein (LRP), sortilin-related receptors (SorLA/LR11), and megalin [13,14]. All these LDLR family members are considered to be endocytosis receptors on the cell surface, which deliver specific ligands to lysosomes to be degraded [15,16,17]. The endocytosis efficiencies of these LDLR family members are different [13]. In addition, these family members are involved in certain pathways that are used to regulate cell adhesion, vesicle trafficking, mitogen-activated protein kinases, neuronal migration, and neurotransmission [18,19,20,21,22,23,24].

VLDLRs, as members of the LDLR family, are multifunctional receptors that are involved in many important physiological processes, such as regulating neuroblast migration in the cerebellum and cerebral cortex, and modulating adipose tissue inflammation [25,26]. To date, the cDNA of *VLDLRs* has mainly been cloned and characterized in vertebrates. Few studies have been conducted on VLDLRs in invertebrates, although some invertebrate genome annotations have been performed. The amphioxus (*Branchiostoma belcheri*) is a marine filter-feeding animal that generally lives in sandy, shallow habitats in tropical and temperate ocean areas [27]. Amphioxi are located at the base in the chordata, and they have been regarded as important in the study of the origin of chordates [28]. For this work, we cloned a new *VLDLR* gene homolog in amphioxi (designated as *AmphiVLDLR*). Additionally, we preliminarily investigated the evolutionary process and molecular function of the *AmphiVLDLR*. Identifying and characterizing the *AmphiVLDLR* gene in *Branchiostoma belcheri* is fundamental to revealing the evolutionary processes that have occurred in this family in chordates. Our results showed an LDLR family member in protochordates and provide new insights into the origin and evolution of chordate LDLRs.

## 2. Materials and Methods

### 2.1. Amphioxus Cultivation

Wild amphioxi (*Branchiostoma belcheri*) were acquired from Zhanjiang (Guangdong province, China) and cultured in seawater (24–25 °C) by using an uninterrupted air circulation system. Before conducting the experiments, we identified the morphological characteristics of the amphioxi using published approaches [29,30,31].

### 2.2. Cloning the Full-Length cDNA of the AmphiVLDLR Gene

Total *Branchiostoma belcheri* RNA was isolated using Trizol reagent (Invitrogen, Carlsbad, CA, USA) according to the manufacturer’s instructions. The cDNA template was synthesized using Moloney murine leukemia virus reverse transcriptase (TaKaRa, Dalian, China). According to the expressed sequence tag of a potential *Branchiostoma floridae* partial *VLDLR* sequence obtained from a library that was constructed in our previous studies [32], two primers were designed to amplify the middle region of *AmphiVLDLR* using PCR (Figure S1). Subsequently, several specific primers were designed to execute 5′ and 3′ RACE experiments (Figure S1). The 5′ and 3′ RACE experiments were conducted using a First Choice ^®^RLM-RACE Kit (Ambion, Austin, TX, USA), according to the manufacturer’s instructions. The cloned fragments were ligated into T-Vector pMD™ 19 plasmids (TaKaRa, Dalian, China) and sequenced. Sequencing results were assembled via DNAMAN to acquire the full-length *AmphiVLDLR* cDNA. Finally, several specific primers were designed to further test the complete CDS, 5′-UTR, ORF, and 3′-UTR regions (Figure S1).

### 2.3. Data Collection, Sequence Alignment, Phylogenetic Analysis, Selective Pressure Analysis, and Gene Annotation

The predicted AmphiVLDLR protein was analyzed by using BLAST programs (http://www.ncbi.nlm.nih.gov/blast/Blast.cgi, accessed on 2 June 2022) [33]. The functional region of the AmphiVLDLR was predicted using the SMART database (http://smart.embl.de/, accessed on 10 June 2022) [34]. The similarity or identity of the known VLDLR sequences were calculated using MatGAT software with the default parameters [35].

To explore the degree of conservation of ten LDLRs, human proteins, including LRP1, LRP1b, LRP2 (megalin), LRP5, LRP6, MegF7, LDLR, VLDLR, ApoER2, and SorLA/LR11, were acquired from the NCBI website (https://www.ncbi.nlm.nih.gov/, accessed on 12 August 2019). These homologous LDLR family proteins were found by using tBLASTn and BLASTp on the genomes of the following species: *Mus musculus*, *Gallus gallus*, *Xenopus laevis*, *Danio rerio*, *Petromyzon marinus*, *Ciona intestinalis*, *Branchiostoma belcheri*, *Strongylocentrotus purpuratus*, *Drosophila melanogaster,* and *Caenorhabditis elegans* (Table S1). All the *Homo sapiens* LDLRs were searched against the Refseq protein data of the above species (except lamprey and amphioxi) (Table S1). These LDLR family sequences in lamprey were searched against the Ensembl database, and those in the amphioxi were searched against the Chinese Lancelet database (http://mosas.sysu.edu.cn/genome/, accessed on 12 August 2019) (Table S1). We filtered all the blast hits, and only eligible sequences (blast score > 150 and length > 50) were examined. Then, all the sequences were searched by using reciprocal blast methods. Additionally, the sequences from the previous step were considered to be homologous genes if the best hit of the initial blast results matched the best hit results of the reciprocal blast. The multiple alignments of LDLR family members were conducted by using the MUSCLE program with default parameters [36]. The phylogenetic tree of the LDLR family proteins was constructed by using phyML software [37]. The signal peptide of the AmphiVLDLR was predicted using SignalP-4.1 (https://services.healthtech.dtu.dk/service.php?SignalP-4.1, accessed on 6 November 2022) [38]. The three-dimensional structures of the *Branchiostoma belcheri* and *Homo sapiens* VLDLR were predicted by using the alphafold2 method in ColabFold (https://colab.research.google.com/github/sokrypton/ColabFold/blob/main/AlphaFold2.ipynb, accessed on 6 November 2022)) [39,40]. The root mean square deviation (RMSD) value was calculated by using VMD to compare the spatial differences in the proteins [41,42]. The genomic structure (exons and introns) analysis was performed by using the Splign platform (https://www.ncbi.nlm.nih.gov/sutils/splign/splign.cgi, accessed on 20 November 2022) [43]. A selective pressure analysis was conducted by using PAML 4.9j [44]. A GO annotation of these VLDLRs was executed by using the InterPro website (https://www.ebi.ac.uk/interpro/, accessed on 2 December 2022), and the KEGG annotation was executed by using eggnog-mapper v2 [45,46].

### 2.4. Real-Time Quantitative PCR Analysis of AmphiVLDLR

To investigate the spatial expression pattern of the *AmphiVLDLR*, the total RNA was extracted from the amphioxi gonads, hepatic cecum, intestines, gills, and muscles (*Branchiostoma belcheri*), and the cDNA was synthesized according to the methods outlined in Section 2.2. The specific *VLDLR* gene primers (5′-GCACAGGACGAACCGCTATC-3′; 5′-GCCACGAGGACGACCAGAG-3′) were designed to execute a quantitative real-time PCR. Additionally, the *β-actin* gene (5′-GCCTCCCTGTCCACCTTCC-3′; 5′-AACTTGCCATCCTTAGCCACTG-3′) of *Branchiostoma belcheri* was used as the internal control. The RT-qPCR was performed using an SYBR^®^ Premix Ex Taq^TM^ kit (TaKaRa, Dalian, China) following the manufacturer’s instructions. All the experiments were conducted in triplicate and were shown in terms of the relative mRNA expression level mean ± SE (n = 4). All the data were processed by using the 2^−∆∆CT^ method, and the gene expression levels in each experiment were visualized in terms of gonads [47].

## 3. Results

### 3.1. Cloning and Characterization of the AmphiVLDLR Gene

**Figure 1:**
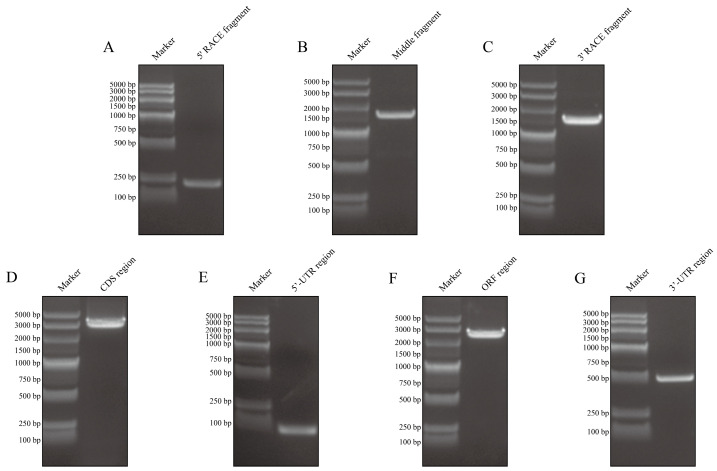
The agarose gel electrophoresis results on *AmphiVLDLR*. The fragments in (**A**–**C**) were obtained after using the rapid amplification of cDNA ends technology. The fragment in (**D**) was obtained using end-to-end PCR. The fragments in (**E**–**G**) were separately obtained when validating the 5′-UTR, ORF, and 3′-UTR regions.

**Figure 2:**
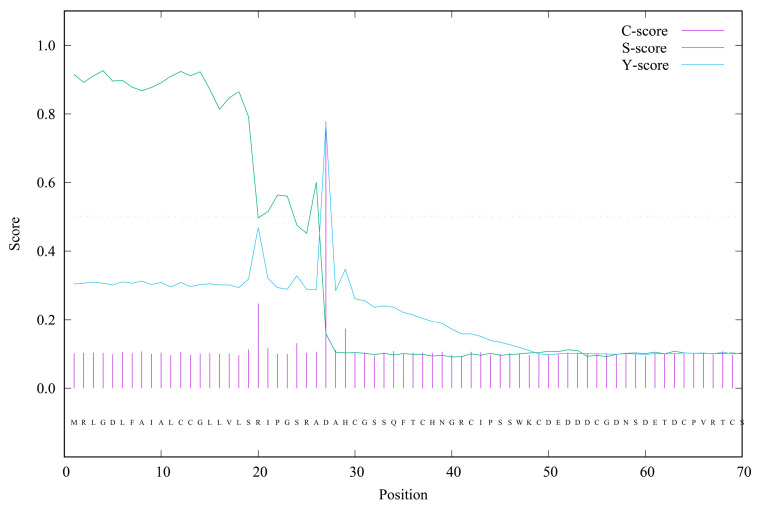
Signal peptide prediction of the AmphiVLDLR protein. The horizontal coordinate represents the 70 amino acids starting from the protein’s N-terminal end. The vertical coordinate represents the score of each index at different amino acid sites. C score: raw cleavage site score; S score: signal peptide score; Y score: combined cleavage site score.

**Figure 3:**
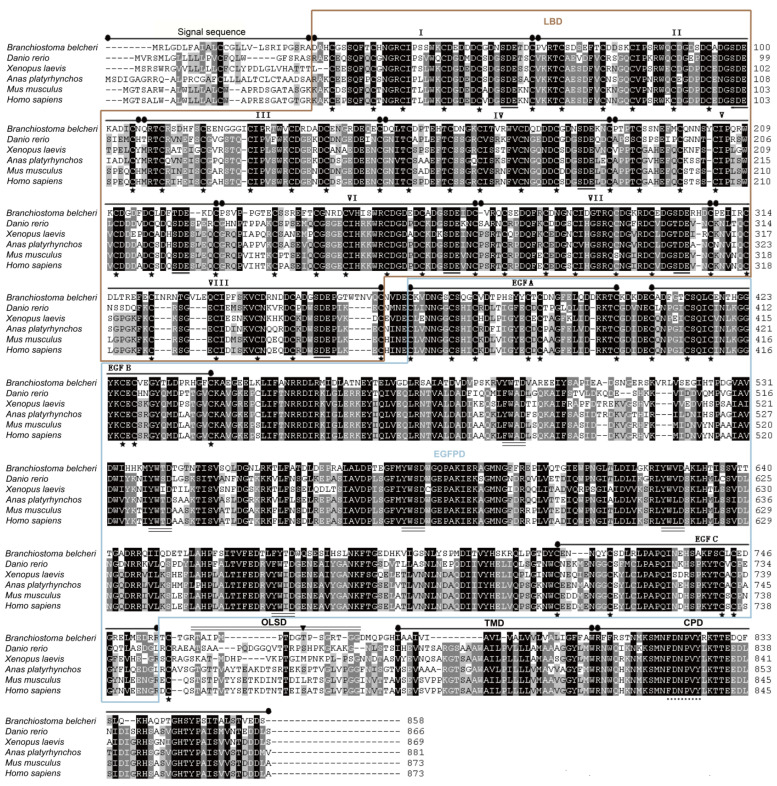
AmphiVLDLR alignment with homologous protein sequences from vertebrates. The eight cysteine-rich repeat domains are numbered I to VIII. The asterisks represent conserved cysteine amino acid residues in the EGFPD (EGF A, B, and C) and cysteine-rich repeat domains. The six cysteine repeats in the LBD may contribute to the formation of a rigid structure in this domain with negatively charged residue clusters (underlined). The YW(T)D sequence was found in multiple tandem repeats. Additionally, the sequences that were predicted to form beta propeller structures are underlined. The OLSD is labeled with double underlining. The conserved FDNPVY motif involved in the receptor endocytosis process is underlined with dots.

**Figure 4:**
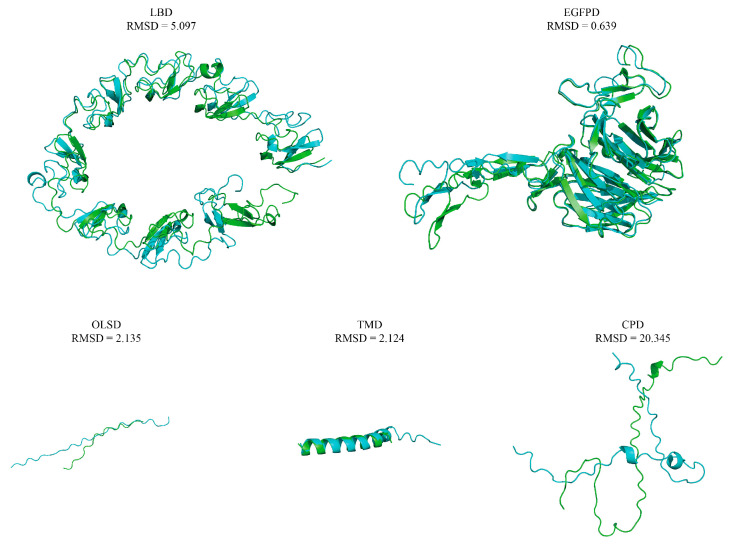
Three-dimensional structure alignment of the *Branchiostoma belcheri* VLDLR and the *Homo sapiens* VLDLR protein domain. Green fragments represent the *Branchiostoma belcheri* functional domain. Blue fragments represent the *Homo sapiens* functional domain.

A 1639 bp middle fragment of the *AmphiVLDLR* gene was amplified from *Branchiostoma belcheri* using RT-PCR. Based on the 1639 bp region, 203 bp (via 5′ RACE) and 1567 bp (via 3′ RACE) fragments were amplified (Figure 1A–C). The full-length *AmphiVLDLR* cDNA (3217 bp) was assembled by overlapping the three cloned fragments. The complete *AmphiVLDLR* cDNA sequence was further tested with an end-to-end PCR reaction (Figure 1D). Additionally, the 5′-UTR, ORF, and 3′-UTR regions were verified using PCR (Figure 1E–G). Ultimately, we confirmed that the complete *AmphiVLDLR* cDNA consisted of a 103 bp 5′-UTR, a 2577 bp ORF, and a 537 bp 3′-UTR. The ORF encoded a putative AmphiVLDLR protein with 858 amino acids. A 26 aa signal peptide was detected (Figure 2), and a relative molecular mass of 93.267 kDa was calculated. Based on the homology alignment of the AmphiVLDLR protein with the VLDLRs of other species, we demonstrated that the AmphiVLDLR was consistent with other VLDLR proteins, including six characteristic structural domains: a cytoplasmic (CPD), transmembrane (TMD), O-linked sugar (OLSD), epidermal growth factor precursor (EGFPD), and ligand-binding domain (LBD) (Figure 3). The spatial alignment results showed that the three-dimensional structure of each functional *Branchiostoma belcheri* VLDLR domain was similar to that of the *Homo sapiens* VLDLR (RMSD_LBD_ = 5.097; RMSD_EGFPD_ = 0.639; RMSD_OLSD_ = 2.135; RMSD_TMD_ = 2.124; RMSD_CPD_ = 20.345) (Figure 4).

**Figure 5:**
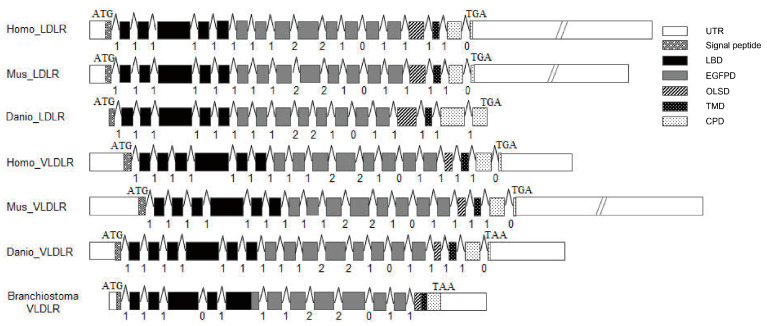
Diagram of the genomic structures of *AmphiVLDLR* and *VLDLR*/*LDLR* genes in vertebrates. The numbers below (∧) represent the phase of the introns. (∧): intron; box: exon.

The results of a genomic sequence analysis showed that the *AmphiVLDLR* gene had 13 exons and 12 introns. The *VLDLR* gene had a conserved exon–intron organization with 19 exons and 18 introns across the vertebrates, even when considering the intron phases and positions (Figure 5). In addition, both the LDLRs and VLDLRs also had a similar gene structure in vertebrates. The only difference was that the VLDLR had 19 exons, whereas the LDLR had 18 exons (Figure 5). The extra exon exactly corresponded to a cysteine-rich repeat sequence in the ligand-binding domain. The intron insertion sites and phases were almost identical in both the VLDLRs and LDLRs, except for the last intron in *Danio rerio* (which was 1 instead of 0). However, in the amphioxi, the insertion positions and phases of the intron underwent large changes, except in the EGFPD region.

The primary AmphiVLDLR structure was compared with other species’ VLDLRs (Figure 3, Table 1). The AmphiVLDLR protein was highly similar to the VLDLR protein from the vertebrates, and the identity degree was different in diverse protein domains (Table 1). The identities of the five characteristic domains were variable. Among them, the identity of LBD, EGFPD, and CPD was relatively high, and that of the other two domains was relatively low (Table 1).

### 3.2. The Presence of LDLR Family Genes in Different Animals

**Figure 6:**
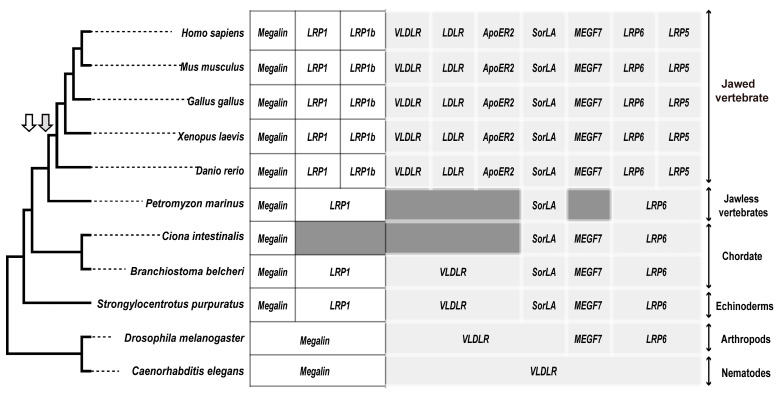
Distribution diagram of the LDLR members in eleven species. The white background corresponds to Clade I of the phylogenetic tree, whereas the light-grey background corresponds to Clade II. The dark grey background corresponds to proteins that were not found in the transcriptome or genome. The white arrows represent gene duplications in Clade I, whereas the grey arrows correspond to duplications in Clade II.

Based on the results of the *VLDLR* gene analysis, we further analyzed the distribution of the ten LDLR family members in the eleven species. Only two LDLR family members (VLDLR and megalin/LRP2) in *Caenorhabditis elegans*, four (VLDLR, megalin/LRP2, MEGF7/LRP4, and LRP6) in *Drosophila melanogaster*, and six in *Strongylocentrotus purpuratus* and *Branchiostoma belcheri* existed, but with a lack of LDLR, LRP1b, LRP5, and ApoER2/LRP8; four (megalin/LRP2, MEGF7/LRP4, LRP6, and SorLA/LR11) family members in *Ciona intestinalis*; four (LRP1, megalin/LRP2, LRP6, and SorLA/LR11) family members in *Petromyzon marinus*; and ten family members of jawed vertebrates (Figure 6) existed.

### 3.3. Alignments and Phylogenetic Analysis of AmphiVLDLR

**Figure 7:**
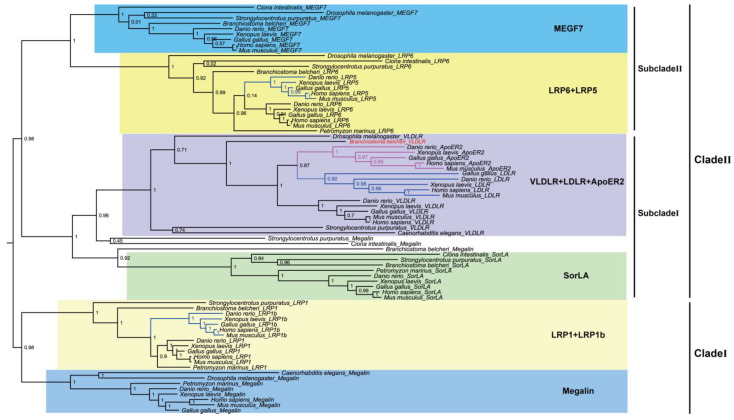
Phylogenetic analysis of LDLR family including AmphiVLDLR. PhyML tree of LDLR family proteins. Blue and pink branches represent replications occurring once and twice, respectively.

To explore the relationship between the AmphiVLDLR and other LDLR family members, representative invertebrate and vertebrate LDLR family sequences (Table S1) were used to construct the evolutionary tree with a maximum likelihood algorithm. All the LDLRs were classified into two clades (Figure 7). Clade I included *megalin, LRP1,* and *LRP1b*. Before *Strongylocentrotus purpuratus* appeared, the *LRP1* gene appeared. Along with the evolution of the species, the duplication of the *LRP1* gene occurred before vertebrates emerged. The *LRP1b* gene was found in jawed vertebrates. Clade II included *VLDLR, LDLR, ApoER2, SorLA, MEGF7, LRP5,* and *LRP6*. Clade II was divided into two subclades. Subclade I included VLDLR, LDLR, ApoER2, and SorLA. Before jawed vertebrates emerged, both *LDLR* and *ApoER2* were generated from the *VLDLR* gene replication. Subclade II included MEGF7, LRP5, and LRP6. The *LRP5* gene was generated via *LPR6* gene replication before jawed vertebrates emerged. The high bootstrap values supported the precision of the topology.

### 3.4. AmphiVLDLR Expression in Different Tissues

**Figure 8:**
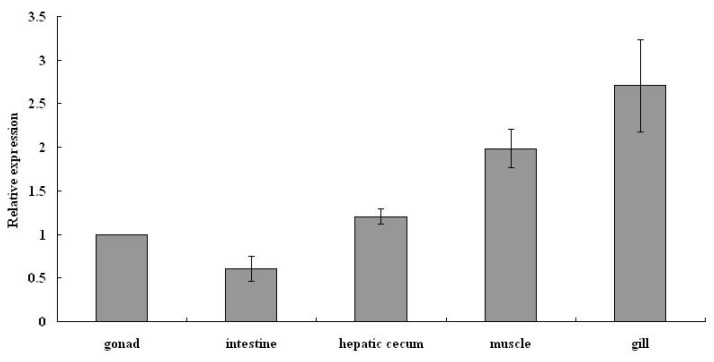
The *AmphiVLDLR* expression level in different tissues.

The spatial expression pattern of the *AmphiVLDLR* in *Branchiostoma belcheri* was explored via RT-qPCR on five different tissues (gonads, hepatic cecum, intestines, gills, and muscles). The results indicated that the *AmphiVLDLR* was ubiquitously expressed in all these tissues (Figure 8). The *AmphiVLDLR* expression level was high in the gills and muscles, moderate in the gonads and hepatic cecum, and low in the intestines (Figure 8).

## 4. Discussion

The LDLR family consists of a group of related receptor proteins with similar structures and functions. The main members are LDLR, VLDLR, ApoER2, LRP1, megalin (LRP2), LRP1b, LRP5, LRP6, MegF7, and SorLA/LR11 [13,14]. Studies on *VLDLR/VLDLR-*like genes are ongoing. Until now, *VLDLRs* have been cloned in mammals, birds, fish, and nematodes, but not in protochordates [1,2,3,4,5,6,7,8]. Amphioxi are considered a species that transformed from invertebrates to vertebrates and represent a basic model for studying the origin and evolution of chordates. Therefore, identifying and characterizing the *AmphiVLDLR* will have high importance for investigating the evolution and function of animal *VLDLR* genes.

### 4.1. AmphiVLDLR Is a New Member of the VLDLR Family

In *Branchiostoma belcheri*, the AmphiVLDLR, with an encoding 3217 bp transcript, was highly homologous to other VLDLR proteins (Figure 3 and Figure 4 and Table 1). Similar to other LDLR family members, the AmphiVLDLR also contained five characteristic domains (Figure 3), and the five domains had high similarities to those for other vertebrates (Figure 3 and Figure 4 and Table 1). The large difference between the *Homo sapiens* and *Branchiostoma belcheri* CPDs (RMSD_CPD_ = 20.345) was due to the high percentage of unstable regions (Figure 4). In the LBD, eight cysteine-rich repeat sequences existed, which was consistent with the VLDLR protein. As shown by the phylogenetic tree, the AmphiVLDLR clustered with the VLDLR + LDLR + ApoER2 invertebrate and vertebrate family members, which was closer to the VLDLRs in invertebrates (Figure 7). This is consistent with the classic evolutionary theory of species. These results support the idea that *AmphiVLDLR* is a type of *VLDLR*.

### 4.2. Evolution of LDLR Family Genes

Based on conservative analysis results, the LDLR family members gradually increased from invertebrate to vertebrate (Figure 6). These new family members may perform different functions. Compared with *Strongylocentrotus purpuratus* and *Branchiostoma belcheri*, *Ciona intestinalis* may have secondarily lost the *VLDLR* and *LRP1 genes*, whereas *Petromyzon marinus* may have secondarily lost the *VLDLR* and *MEGF7* genes (Figure 6). The *megalin* gene from *Strongylocentrotus purpuratus*, *Branchiostoma belcheri*, and *Ciona intestinalis* did not cluster with other megalin family members. This may be due to the three sequences having extra molecular functions compared with the sequences in the megalin family of other species, or the genome annotation was incomplete in these three species.

Combined with the results of the conservative analysis and phylogenetic tree (Figure 6 and Figure 7), we found that the LDLR family members experienced duplication and deletion events during evolution. When transitioning from invertebrates to vertebrates, the LDLR family members experienced duplications three times, i.e., LRP1b was duplicated from LRP1, ApoER2 and LDLR were duplicated from VLDLR, and LRP5 was duplicated from LRP6. These gene-generating events may be related to two rounds of whole-genome duplication in vertebrates [48]. The *VLDLR*, as one of the ancient LDLR family members, must have important biological functions; thus, identifying the *AmphiVLDLR* gene will be helpful in uncovering the function and evolution of the LDLR family in different species.

Based on the phylogenetic tree, we further compared the AmphiVLDLR with the LDLRs and VLDLRs of vertebrates. Among all the analyzed LDLR family proteins, the *LDLR* and *VLDLR* gene structures were very similar in vertebrates except for the insertion phase of the last intron from the LDLR in *Danio rerio*. The sequence similarities and the gene structures indicated that the LDLRs and VLDLRs may have originated from a common ancestral gene. The differences in the intron numbers of the invertebrate VLDLR homolog genes suggest that some introns may have been inserted into the VLDLR homologues during invertebrate evolution [49,50]. Compared with the LDLR and VLDLR in vertebrates, the *AmphiVLDLR* gene structure is very different. Therefore, we speculate that intron insertion events also occurred in vertebrates. The intron gain in vertebrates may be related to the functional elements of introns. Intron insertion is probably relevant for multiple functions, including encoding untranslated RNA, alternative splicing, and enhancing a normal or necessary mRNA transcription level and function at the DNA level [51,52,53,54,55,56]. An unknown mechanism whereby introns are appropriately added needs to be further investigated in the future to clarify this phenomenon.

### 4.3. VLDLR Tissue Expression Pattern

To investigate the function of the *AmphiVLDLR*, we detected its expression level in various tissues. *VLDLR* mRNA is mainly present in the heart, skeletal muscles, and adipose tissues in human [10]. Mouse VLDLRs are most abundant in the brain and are especially highly expressed in skeletal muscles and the heart. The existence of high-level free lipids in these tissues supports the idea that VLDLRs play vital roles in fatty acid and cholesterol metabolism [57]. The *AmphiVLDLR* mRNA was present in all tissues and was abundant in the gills and muscles and deficient in the gonads and intestines (Figure 8). The relatively abundant *AmphiVLDLR* mRNA distribution in the muscles was similar to that in mice and humans, which may also be related to cholesterol and fatty acid metabolism. The GO annotation indicated that a major function of the AmphiVLDLR was calcium-ion binding (Table S2). Calcium ions perform multiple metabolic functions in muscles, and gills are the major organ for active calcium ion uptake, which has a high importance for the maintenance of calcium ion homeostasis [58]. The high expression level of the *Branchiostoma belcheri VLDLR* gene in the muscles and gills demonstrates that the *AmphiVLDLR* has similar conserved functions. Although both the GO and KEGG annotations showed that the functions of the VLDLR family were conserved among different species (Tables S2 and S3), the results of the selection pressure analysis demonstrated that the *VLDLR* gene in amphioxi had numerous positive selection sites (Table S4). These results implied that the *AmphiVLDLR* not only performs conserved functions compared with other *VLDLR* genes, but it may also have unique biological functions after undergoing adaptive evolution.

## 5. Conclusions

In summary, we identified the AmphiVLDLR as a new member of the VLDLR family and provided insight into the evolution of the LDLR family members from invertebrates to vertebrates.

## Figures and Tables

**Table 1:** Identity (%) of modular domains between AmphiVLDLR and VLDLRs of other species with different evolutionary statuses.

Protein	Overall Protein	LBD	EGFPD	OLSD	TMD	CPD
*Danio rerio* VLDLR	45.8	49.9	47.1	19.4	26.5	46.3
*Xenopus laevis* VLDLR	44.5	48.7	45.4	14.7	17.6	48.1
*Anas platyrhynchos* VLDLR	45.9	49.0	49.0	20.0	24.2	48.1
*Mus musculus* VLDLR	45.5	48.7	48.1	22.5	20.6	50.0
*Homo sapiens* VLDLR	45.3	48.7	47.8	20.5	20.6	50.0

The numbers represent the identity (%) of corresponding species respecting to AmphiVLDLR.

## Data Availability

Not applicable.

## Supplementary Materials

The following supporting information can be downloaded at: https://www.mdpi.com/article/10.3390/ani13132193/s1, Figure S1: The schematic diagram of PCR primers and corresponding regions; Table S1: The information of LDLR family genes and proteins; Table S2: GO annotations of the VLDLR family members; Table S3: KEGG annotations of the VLDLR family members; Table S4: Selective pressure analysis (branch-site model) of *AmphiVLDLR.*
