# Supplementary material for: Identification and Characterization of the Very-Low-Density Lipoprotein Receptor Gene from Branchiostoma belcheri: Insights into the Origin and Evolution of the Low-Density Lipoprotein Receptor Gene Family
Source: Animals (Basel). 2023 Jul 4;13(13):2193. doi: 10.3390/ani13132193 (PMC10339998; doi:10.3390/ani13132193)
Supplement: Supplementary file 1 [file animals-13-02193-s001.zip › Figue S1.pdf]

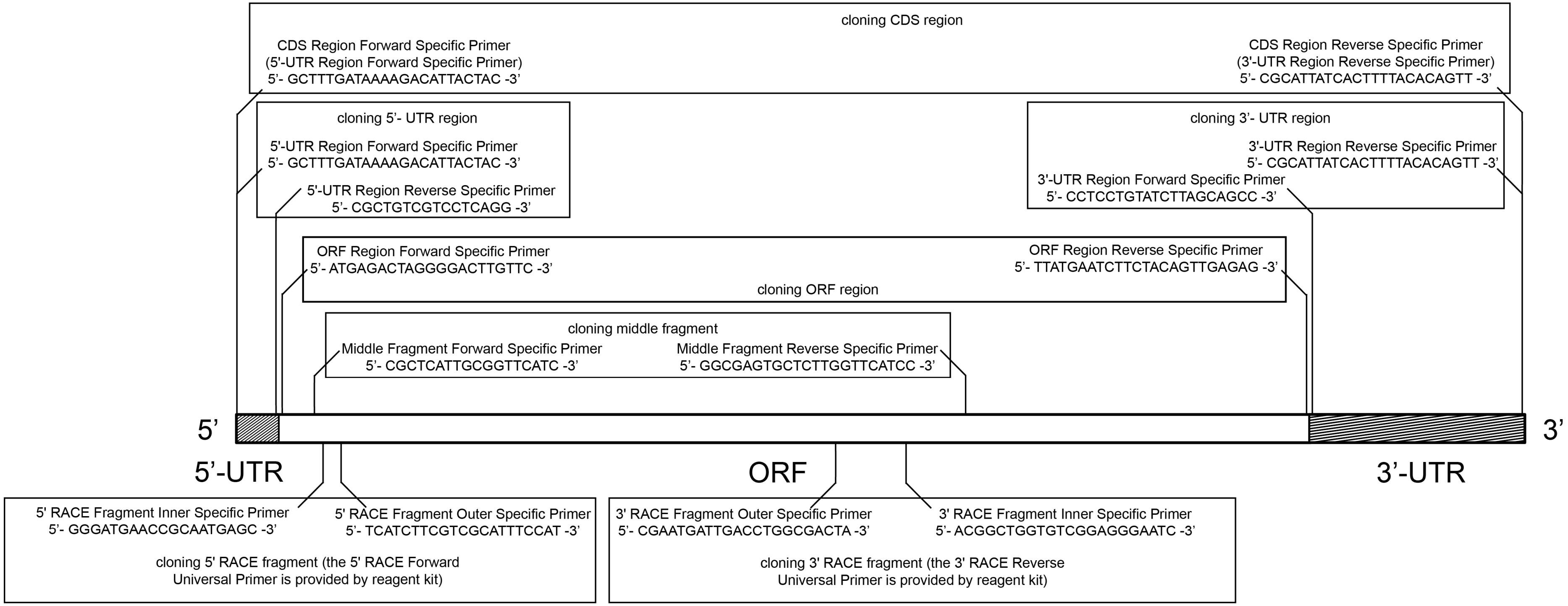

Figure S1. The schematic diagram of PCR primers and corresponding regions. Blank box represents the *AmphiVLDLR* ORF region; Grid boxes represent the *AmphiVLDLR* 5'-UTR and 3'-UTR region. Middle Fragment Forward Specific Primer and Middle Fragment Reverse Specific Primer work for amplifying the *AmphiVLDLR* middle fragment; 5' RACE Fragment Outer Specific Primer and 5' RACE Fragment Inner Specific Primer work for amplifying the *AmphiVLDLR* 5' RACE fragment; 3' RACE Fragment Outer Specific Primer and 3' RACE Fragment Inner Specific Primer work for amplifying the *AmphiVLDLR* 3' RACE fragment; CDS Region Forward Specific Primer and CDS Region Reverse Specific Primer work for amplifying the *AmphiVLDLR* CDS region; 5'-UTR Region Forward Specific Primer and 5'-UTR Region Reverse Specific Primer work for amplifying the *AmphiVLDLR* 5'-UTR region; ORF Region Forward Specific Primer and ORF Region Reverse Specific Primer work for amplifying the *AmphiVLDLR* ORF region; 3'-UTR Region Forward Specific Primer and 3'-UTR Region Reverse Specific Primer work for amplifying the *AmphiVLDLR* 3'-UTR region.
