# Supplementary material for: Identification and Characterization of the Very-Low-Density Lipoprotein Receptor Gene from Branchiostoma belcheri: Insights into the Origin and Evolution of the Low-Density Lipoprotein Receptor Gene Family
Source: Animals (Basel). 2023 Jul 4;13(13):2193. doi: 10.3390/ani13132193 (PMC10339998; doi:10.3390/ani13132193)
Supplement: Supplementary file 1 [file animals-13-02193-s001.zip › Table S1.pdf]

Table S1 The information of LDLR family genes and proteins

Common name	Scientific name	protein ID.										Resource
		LDLR	VLDLR	ApoER2/LRP8	MEGF7/LRP4	LRP1	LRP1b	Megalin/LRP2	LRP5	LRP6	SorLA/LR11	
Human	*Homo sapiens*	NP_000518	NP_003374.3	NP_004622.2	NP_002325	NP_002323	NP_061027	NP_004516	NP_002326	NP_002327.2	NP_003096	NCBI
House mouse	*Mus musculus*	NP_034830	NP_038731	NP_001074395	NP_766256	NP_032538	NP_443737	NP_001074557	NP_032539	NP_032540	NP_035566	NCBI
Chicken	*Gallus gallus*	NP_989783	NP_990560	NP_990517	XP_421114	NP_990573	XP_422146	XP_004942822	NP_001012915	XP_417286	NP_001292089	NCBI
African clawed frog	*Xenopus laevis*	NP_001081290	NP_001084168	XP_004914072	XP_012809498	XP_002939758	XP_012808483	XP_002936802	XP_002941689	XP_012814842	XP_012822697	NCBI
Zebrafish	*Danio rerio*	NP_001025454	NP_957217	XP_005166314	XP_009301924	XP_005162276	XP_009303127	NP_001181916	NP_001170929	NP_001128156	XP_005157607	NCBI
Sea lamprey	*Petromyzon marinus*	None	None	None	None	ENSPMAP00006907	None	ENSPMAP00004159	None	ENSPMAP00009333	ENSPMAP00000003785	Ensemble
Vase tunicate	*Ciona intestinalis*	None	None	None	XP_009858405	None	None	XP_009861968	None	XP_002128714	XP_009859467	NCBI
Belcher's lancelet	*Branchiostoma belcheri*	None	Clone	None	BbP_208230F	BbP_019940F	None	tblastN,Sc0000062:679737-680741+wise2	None	BbP_195320F	BbP_205600R	Chinese Lancelet Genome
Purple sea urchin	*Strongylocentrotus purpuratus*	None	XP_011678205	None	XP_011663978	XP_011680982	None	XP_011683968	None	XP_795095	XP_011674618	NCBI
Fruit fly	*Drosophila melanogaster*	None	NP_001097926	None	NP_001162769	None	None	NP_001096924	None	NP_524737	None	NCBI
Nematode	*Caenorhabditis elegans*	None	NP_495387	None	None	None	None	NP_492127	None	None	None	NCBI
