# Supplementary material for: Identification and Characterization of the Very-Low-Density Lipoprotein Receptor Gene from Branchiostoma belcheri: Insights into the Origin and Evolution of the Low-Density Lipoprotein Receptor Gene Family
Source: Animals (Basel). 2023 Jul 4;13(13):2193. doi: 10.3390/ani13132193 (PMC10339998; doi:10.3390/ani13132193)
Supplement: Supplementary file 1 [file animals-13-02193-s001.zip › Table S2.pdf]

Table S2 GO annotations of the VLDLR family members

Gene	Biological Process	Molecular Function	Cellular Component
Homo sapiens VLDLR	None	Protein binding (GO:0005515)<br>Calcium ion binding (GO:0005509)	None
Mus musculus VLDLR	None	Protein binding (GO:0005515)<br>Calcium ion binding (GO:0005509)	None
Xenopus laevis VLDLR	None	Protein binding (GO:0005515)<br>Calcium ion binding (GO:0005509)	None
Danio rerio VLDLR	None	Protein binding (GO:0005515)<br>Calcium ion binding (GO:0005509)	None
Branchiostoma belcheri VLDLR	None	Protein binding (GO:0005515)<br>Calcium ion binding (GO:0005509)	None
