# Supplementary material for: Identification and Characterization of the Very-Low-Density Lipoprotein Receptor Gene from Branchiostoma belcheri: Insights into the Origin and Evolution of the Low-Density Lipoprotein Receptor Gene Family
Source: Animals (Basel). 2023 Jul 4;13(13):2193. doi: 10.3390/ani13132193 (PMC10339998; doi:10.3390/ani13132193)
Supplement: Supplementary file 1 [file animals-13-02193-s001.zip › Table S3.pdf]

Table S3 KEGG annotations of the VLDLR family members

**Gene**	**KEGG_Pathway**
Homo sapiens VLDLR	ko04144, ko04913, ko04925, ko04927, ko04934, ko04976, ko04979, ko05145, ko05160
Mus musculus VLDLR	ko04144, ko04913, ko04925, ko04927, ko04934, ko04976, ko04979, ko05145, ko05160
Xenopus laevis VLDLR	ko04144, ko04913, ko04925, ko04927, ko04934, ko04976, ko04979, ko05145, ko05160
Danio rerio VLDLR	ko04144, ko04913, ko04925, ko04927, ko04934, ko04976, ko04979, ko05145, ko05160
Branchiostoma belcheri VLDLR	ko04144, ko04913, ko04925, ko04927, ko04934, ko04976, ko04979, ko05145, ko05160
