# Supplementary material for: Identification and Characterization of the Very-Low-Density Lipoprotein Receptor Gene from Branchiostoma belcheri: Insights into the Origin and Evolution of the Low-Density Lipoprotein Receptor Gene Family
Source: Animals (Basel). 2023 Jul 4;13(13):2193. doi: 10.3390/ani13132193 (PMC10339998; doi:10.3390/ani13132193)
Supplement: Supplementary file 1 [file animals-13-02193-s001.zip › Table S4.pdf]

Table S4 Selective pressure analysis (branch-site model) of *AmphiVLDLR*

**Model**	**df**	**-lnL**	**2△lnL**	***P* value**	**Parameter**	**Positive selection site**
MA0		441057.092456			P_0=0.67877, P_1=0.04715, P_{2a}=0.25629, P_{2b}=0.01780, ω_0=0.06719, ω_1=1.00000, ω_2=1.00000	
MA	1	441040.255609	33.673694	6.517614e-09	P_0=0.65489, P_1=0.04559, P_{2a}=0.28003, P_{2b}=0.01949, ω_0=0.06747, ω_1=1.00000, ω_2=8.60735	22 P, 36 T, 56 G, 59 S, 124 I, 136 A, 142 R, 149 Q, 157 H, 173 Q, 180 N, 190 T, 192 S, 203 Y, 218 L, 220 F, 241 T, 272 Q, 274 S, 275 E, 313 R, 316 L, 330 L, 344 D, 361 C, 367 K, 376 G, 380 T, 382 H, 383 S, 385 Y, 402 K, 404 K, 410 F, 437 R, 440 F, 448 L, 449 K, 184 T, 513 R, 514 S, 517 R, 536 H, 552 Q, 601 F, 607 Q, 636 S, 637 S, 639 T, 640 T, 647 Q, 651 D, 670 Y, 681 L, 687 E, 689H, 698 S, 708 K, 721 Q, 722 Y, 743 L, 745 E, 751 M, 752 E, 760 R, 763 I, 774 R, 777 G, 779 M, 784 I, 802 I, 809 F, 810 F, 828T, 829 T, 832 Q

Screening condition for positive selection sites: $P < 0.05$ by LRT test of BEB analysis
